# Supplementary material for: “…this could be a noble idea and a game changer.” The potential of a dual prevention pill for HIV and pregnancy prevention among adolescent girls and young women in Zimbabwe
Source: PLOS Glob Public Health. 2025 Aug 28;5(8):e0005071. doi: 10.1371/journal.pgph.0005071 (PMC12393725; doi:10.1371/journal.pgph.0005071)
Supplement: Attachment — Submitted filename: Response to Reviewers_Rebuttal Letter 18Oct24.pdf [file pgph.0005071.s003.pdf]

University of Zimbabwe
Clinical Trials Research Centre

Administrative Office
15 Phillips Avenue, Belgravia, Harare, Zimbabwe
Tel: +263-24-2704890/2704920, Fax: +263-24-2704897

Research Office
10 Routledge Street, Milton Park, Harare, Zimbabwe
Tel: +263-24-252299/27911374, Fax: +263-24-2704897

October 18, 2024

The Editors
PLOS Global Public Health

Dear Editors,

I am pleased to submit our revised research article titled, **"…this could be a noble idea and a game changer." The potential of a dual prevention pill for HIV and pregnancy prevention among adolescent girls and young women in Zimbabwe"** for consideration at PLOS Global Public Health.

We value the reviewer's interest in our work and we are grateful for their insightful feedback. We believe the revisions have resulted in a substantially improved manuscript. We have addressed the comments point-by-point below. The comments are copied in the table below alongside our responses.

Reviewer Comments	Response
This is a well written article evaluating the opinions of users (adolescent girls and young women) and providers (health care providers) in Zimbabwe on the introduction of a dual prevention pill (DPP). The study showed that the DPP was welcomed by both providers and users but there were some concerns about potential additive side effects, lack of protection against other STIs and needing to distinguish the packaging from antiretrovirals to minimize stigma.	Thank you for your kind comments and for your helpful suggestions on how we can improve the manuscript.
**Abstract**	
It would be beneficial to include the sample size in the abstract so the readers can know upfront on the scale of the study.	Thank you for this suggestion. We have revised the abstract in line with your suggestion.
**Introduction**	
Line 90-93 - this sentence, "Guided by the framework.... and intent to use" may belong better	Thank you for bringing this to our attention. We have moved the sentence to the methods section.

in the methods.	
**Results**	
Table 1 - please include the range for the ages. Given that the group of AGYW were stratified into 2 age groups (16-19 years and 20-24 years), can you include the proportion < 20 and > 20 years in the table? Of the 32 AGYW who participated, it is not clear if this was equally divided between the 2 age strata	Thank you for your comment and suggestion. We have included the age ranges and proportion in table 1.
Line 186 - can you provide the % for the sentence "Most (??%) of the HCP..."	Thank you for bringing this to our attention. We have provided the percentage in line with your suggestion.
**Discussion**	
Line 520-521 - please include a reference for the other end user studies that indicate there is a need for MPTs.	Thank you for catching this oversight, we have included the reference as per your suggestion.
Line 559-560 – please rephrase the sentence. Something seems to be missing from the sentence.	Thank you for bringing this to our attention. We rephrased the sentence to make it more coherent.
**References**	
Please provide a doi for ref 6, 7, 22, 27 and 33	Thank you for bringing this to our attention. We have provided the doi for the references.
Ref 17 - provide date accessed	Thank you for catching this oversight, we have provided the date accessed.
Ref 18 - there seems to be newer guidelines published in 2024 - MMWR 2024; 73(4);1–126 - if appropriate, the newer guidelines should be referenced. if the 2020 publication is still appropriate, provide the end pages, i.e., 405-410	Thank you for bringing this to our attention. It seems the 2020 publication is still applicable for our citation. We have provided the end pages for the 2020 publication.

Ref 26 - provide publisher and place pf publication	Thank you for catching this oversight, we have provided the publisher and place of publication.
Ref 28 - provide date accessed	Thank you for catching this oversight, we updated the citation and referencing a paper, which we had already cited, instead of the fact sheet.

Thank you for your consideration. Please feel free to reach out to me if you have any questions.

Sincerely,
Adlight Dandadzi, BSc Hons Sociology
Corresponding Author
University of Zimbabwe - Clinical Trials Research Centre
adandadzi@uz-ctrc.org
