# Supplementary material for: “…this could be a noble idea and a game changer.” The potential of a dual prevention pill for HIV and pregnancy prevention among adolescent girls and young women in Zimbabwe
Source: PLOS Glob Public Health. 2025 Aug 28;5(8):e0005071. doi: 10.1371/journal.pgph.0005071 (PMC12393725; doi:10.1371/journal.pgph.0005071)
Supplement: Attachment — Submitted filename: PGPH-D-24-01238R1_response to reviewers.docx [file pgph.0005071.s004.docx]

**Response to Reviewer Comments for PGPH-D-24-01238R1**

**July 10, 2025**

Reviewer #2: Thank you for the opportunity to review the manuscript, “…this could be a noble idea and a game changer.” The potential of a dual prevention pill for HIV and pregnancy prevention among adolescent girls and young women in Zimbabwe”. The paper is well-written and presented, and I have only minor comments.
*Thank you, kindly see our responses below.*

- Why were IDIs not done with the end users? Could the authors give some rationale why only FGDs were done for end-users and IDIs for HCPs?

*Thanks for your question. In asking about this hypothetical product, we felt that FGDs were a more appropriate methodology to use in this formative study, to allow participants to reflect and build on each other’s perspectives. We conducted IDIs with HCPs because we were trying to capture perspectives from different cadre of HCPs. Additionally, we interviewed HCPs at a time and location that was most convenient to them without distracting from their heavy workload; it would have been logistically challenging to gather HCPs for a FGD.*

- How many participants per FGD on average? I think the authors give only aggregate number (32 for total 4 FGDs).

*There were 6-8 participants per FGD. We have added this to line 174 in the manuscript.*

- Line 189: Was there a quantitative survey? Or is the percentage based on the contents from IDIs? I wouldn't put percentage, if these data from IDIs.

*Thank you, the statement reflects qualitative data. We have adjusted the statement.*
